# Cross-Jurisdictional Transmission of Mycobacterium tuberculosis in Maryland and Washington, D.C., 1996–2000, Linked to the Homeless

**DOI:** 10.3201/eid0811.020245

**Published:** 2002-11

**Authors:** Monica Lathan, Leonard Ntaate Mukasa, Nancy Hooper, Jonathan Golub, Nancy Baruch, Donna Mulcahy, William Benjamin, Wendy A. Cronin

**Affiliations:** * Maryland Department of Health and Mental Hygiene, Baltimore, Maryland, USA; † American Public Health Association, Washington, D.C., USA; ‡ Alabama Department of Health, Montgomery, Alabama, USA; § University of Alabama at Birmingham, Birmingham, Alabama, USA

**Keywords:** tuberculosis, molecular epidemiology, cluster, homelessness

## Abstract

From 1996 to 2000, 23 Maryland and Washington, D.C., tuberculosis cases were identified in one six-band DNA cluster. Cases were clustered on the basis of their Mycobacterium tuberculosis isolates. Medical record reviews and interviews were conducted to identify epidemiologic linkages. Eighteen (78%) of the 23 case-patients with identical restriction fragment length polymorphism patterns were linked to another member; half the patients were associated with a Washington, D.C., homeless shelter. Molecular epidemiology defined the extent of this large, cross-jurisdictional outbreak.

A rise in homelessness in particular and poverty in general partially accounted for the resurgence of tuberculosis (TB) in the United States from 1984 to 1992 (1,2). In Maryland, as part of the National Tuberculosis Genotyping and Surveillance Network activities, population-based DNA fingerprinting of Mycobacterium tuberculosis isolates from culture-positive patients was conducted from January 1996 through December 2000. Selected Washington, D.C., isolates from TB patients with suspected or known homelessness were DNA fingerprinted as early as 1996. The Washington, D.C. TB Control staff determined suspected or known homelessness from information contained in case histories and medical records. An interjurisdictional investigation was conducted among homeless persons in Washington, D.C., and Maryland to establish epidemiologic linkages.

## The Study

Standard methods were used for IS6110 restriction fragment length polymorphism (RFLP) analysis of M. tuberculosis isolates (3). A cluster was defined as a group of two or more cases with a matching DNA fingerprint pattern (+/-1 band). Spoligotyping (secondary typing) was performed on all clustered strains having six or fewer copies of IS6110 (4). Medical record reviews and interviews were conducted for all clustered cases to identify connections. We used chi-square and Fisher exact tests to compare demographic and clinical characteristics between the homeless and nonhomeless groups.

A homeless person was defined, at the time a case was reported to the health department, as a person who lacked a fixed, regular, and adequate nighttime residence within the past year or a person who gave a history of homelessness in the recent past (1–5 years). Alcoholism and drug use were routinely documented on the TB case report form. Alcohol abuse was defined as patient-reported alcoholism or disclosure of excessive alcohol use. Recent drug use was defined as injecting or noninjecting drug use within the past year.

From January 1996 through December 2000, nearly all (99 % or 1,181/1,191) of the culture-positive isolates from Maryland were DNA fingerprinted. Since the District of Columbia was not a network sentinel site in the genotyping network, only 29 (9%) of the 318 M. tuberculosis isolates from culture-positive Washington, D.C., cases were fingerprinted (only those for outbreak investigations). Maryland Cluster A6 (Centers for Disease Control and Prevention [CDC] designations 00104 and 00645) consisted of 23 case isolates; 15 (65%) A6 case-patients were residents of Maryland, and the remaining 8 (35%) were District of Columbia residents. Spoligotypes (CDC designation 3) were identical for all 23 isolates. The first known case in Cluster A6 was reported in March 1996; 22 subsequent cases occurred through November 2000 (Figure). All patients were born in the United States and were African Americans. Homelessness was documented for 14 (61%) of the 23 patients. Eighty-seven percent had acid-fast bacilli smear-positive sputum, and 52% had pulmonary, cavitary disease. Other TB risk factors included alcohol abuse (52%), HIV infection (39%), and drug use (22%). Nonhomeless persons differed from homeless persons because they were more likely to be women (p<0.02) and were less likely to have identified risk behaviors. Nonhomeless patients were similar to the homeless in the proportion of cases with pulmonary cavities (Table).

**Figure:**
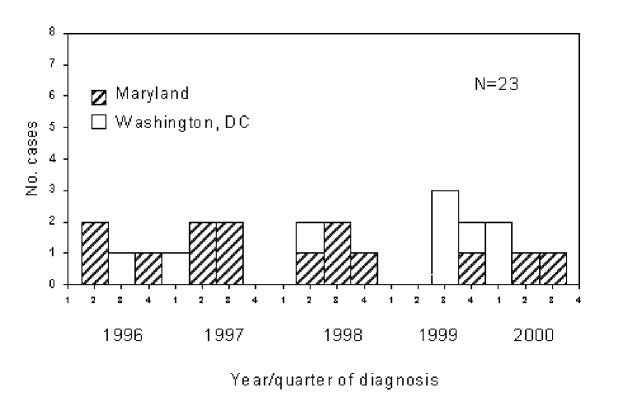
Cluster A6 tuberculosis cases, Maryland and Washington, D.C.

**Table:** Selected characteristics among homeless and nonhomeless clustered tuberculosis case-patients, Maryland and Washington, D.C.a,b

Characteristics	Homeless No. (%)	Non-homeless No. (%)	p valuec
Gender			
Male	13 (92.8)	4 (44.4)	0.02
Female	1(7.2)	5 (55.6)	
Median age in years (range)	42 (27–57)	40 (23–61)	0.97
Substance abuse (IV or non-IV)			
Yes	5 (35.7)	0 (0.0)	
No	9 (64.3)	9 (100.0)	0.11
Alcohol abuse			
Yes	9 (64.3)	3 (33.3)	0.21
No	5 (35.7)	6(66.7)	
HIV status			
Positive	8 (57.1)	1 (11.1)	0.16
Negative	6 (42.9)	6 (66.7)	
Unknown	0 (0.0)	2 (22.2)	
Smear for AFB			
Positive	14 (100.0)	6 (66.7)3	0.05
Negative	0 (0.0)	(33.3)	
Pulmonary cavities			
Present	7 (50.0)	5 (55.6)	1.00
Absent	7 (50.0)	4 (44.4)	
Mental illness			
Yes	4 (28.6)	1 (11.1)	0.61
No	10 (71.4)	8 (88.9)	
Died			
Yes	4 (28.6)	1 (11.1)	0.61
No	10 (71.4)	8 (88.9)	

aNA, not applicable; AFB, acid-fast bacilli. bWashington, D.C., cases were preselected on the basis of a homeless history except for case in the nurse. cFisher exact tests were used to compare demographic and clinical characteristics between the homeless and nonhomeless groups.

Most cases (78% or 18/23) in Cluster A6 had epidemiologic connections. Nine (50%) of the connected case-patients were directly linked to a large homeless shelter in Washington, D.C. Six case-patients were connected by time and place on the basis of histories of homelessness, socializing with homeless persons, caring for a homeless patient, and sharing boarding or transitional houses. The remaining three were rural nonhomeless case-patients connected by workplace and social links (e.g., drinking); however, they had no other known links with other cluster members. More than one third of the relationships were identified only after the DNA cluster investigation.

In late 1998, active disease caused by the same strain was diagnosed in a nurse who had cared for two hospitalized persons (cluster case-patients), a nonhomeless Maryland resident in 1997 and a homeless Washington, D.C., resident in 1998. Both hospitalized persons were highly infectious with sputum smear-positive and cavitary disease. The nurse cared for the first patient before the patient’s diagnosis and subsequent isolation and cared for the second patient only during isolation. Because the second patient reportedly often removed his mask and left his isolation room, we could not determine definitively which case-patient was the source of the nurse’s infection.

Our molecular epidemiology study identified TB transmission between homeless and nonhomeless settings in Maryland and provided an opportunity to assess transmission between the state and adjacent Washington, D.C. Population-based molecular epidemiologic studies consistently demonstrate that TB transmission is geographically localized in one or two adjoining jurisdictions (5–8). We are aware of only two DNA-confirmed instances of TB transmission between states (9,10). Interestingly, the Cluster A6 strain exactly matched that seen in a large outbreak among the homeless in Syracuse, New York (J Driscoll, pers. comm.) (11). The Syracuse outbreak began with a single, highly infectious case that was reported in 1992. We do not know when this strain appeared in Maryland because statewide genotyping was not available in our area before 1996. Further investigation and consultation with Syracuse health department staff showed no additional epidemiologic links between the two states.

The Cluster A6 strain was not identified in isolates analyzed in two other sentinel sites in the TB genotyping network (Massachusetts and New Jersey) or more recently in New York City homeless or nonhomeless outbreaks (J. Driscoll, pers. commun.). This observation suggests that the existing links may not be among immediate neighbors but may be more far-reaching. As disease incidence continues to decline and programs consolidate into regional offices (12), the expanded use of molecular epidemiology will prove increasingly valuable in TB investigations.

Even with expanded (or regional) genotyping of isolates, an active relationship between jurisdictions remains essential to prevent transmission or progression to active disease in patients and their contacts within a region. To investigate Cluster A6, Maryland and Washington, D.C., TB control staff held frequent meetings and teleconferences to review data on RFLP patterns, case characteristics and locations, and contact information. Local health department personnel in Washington, D.C., and adjacent Maryland counties routinely share contact data on as many as half of their cases (TB Programs, Washington, D.C., and Maryland, unpub. data, 2002).

Whether the Cluster A6 strain was introduced recently or was a result of prior TB in the Washington, D.C., homeless population is not unknown. The underlying tuberculin skin-test (TST) positivity was unknown for most case-patients, and only one person had a documented TST conversion. However, persons at high risk for TB, particularly those who were homeless or had HIV infection, are at increased risk for exogenous reinfection (13,14). Homelessness, along with other TB risk factors, can make treatment a daunting task.

Cases reported in 2000 indicate that this large outbreak was not controlled effectively (Figure). Three cases with this strain were also reported in 2001—the most recent in October. The ongoing appearance of patients indicates that TB in the homeless continues to be a challenge in the region. Although 78% of the case connections were found in this difficult-to-reach population, one third of these linkages were identified only after DNA fingerprinting of the M. tuberculosis isolates. More thorough contact investigations would not likely have established connections. Cofactors such as substance abuse, mental illness, and HIV infection further complicate the likelihood of obtaining reliable histories and contact reports. Although Cluster A may have eventually been traced to the homeless shelter, the magnitude of the outbreak might have never been realized without the inclusion of District of Columbia cases in our DNA fingerprinting sample (regionalization). Universal M. tuberculosis genotyping in Washington, D.C., would likely have shown additional cases clustered among the homeless and nonhomeless and further defined the extent of the outbreak.

## Conclusions

Molecular epidemiology showed unsuspected TB transmission across jurisdictional borders and transmission involving the homeless and the nonhomeless populations. Investigation of this large interjurisdictional cluster required close collaboration between the Maryland and Washington, D.C., TB Control Programs. As disease incidence continues to decline, regionalizing program efforts with associated M. tuberculosis genotyping will be essential to detect and prevent ongoing disease transmission, particularly in difficult-to-reach populations.
